# Early platelet level reduction as a prognostic factor in intensive care unit patients with severe aspiration pneumonia

**DOI:** 10.3389/fphys.2023.1064699

**Published:** 2023-03-07

**Authors:** Li-Na Wang, Dai-Kun He, Yi-Ru Shao, Jiang Lv, Peng-Fei Wang, Ying Ge, Wei Yan

**Affiliations:** ^1^ Department of General Practice, Jinshan Hospital, Fudan University, Shanghai, China; ^2^ Center of Emergency and Intensive Care Unit, Jinshan Hospital, Fudan University, Shanghai, China; ^3^ Medical Research Centre for Chemical Injury, Emergency and Critical Care, Jinshan Hospital, Fudan University, Shanghai, China

**Keywords:** prognosis, platelet, mortality, intensive care units, aspiration pneumonia, severe pneumonia, risk factors

## Abstract

**Introduction:** This study investigates risk factors underlying the prognosis of severe aspiration pneumonia (SAP) in intensive care unit (ICU) patients and attempts to provide early prognosis reference for clinical tasks.

**Methods:** Patients diagnosed with SAP and admitted to the ICU of Jinshan Hospital, Fudan University, Shanghai, China, between January 2021 and December 2021 were recruited in this retrospective cohort study. Clinical data on a patient’s general condition, underlying diseases, laboratory indicators, and 90-day outcomes (survival or death) were recorded.

**Results:** Multivariate logistic regression analysis showed that a low platelet count was an independent risk factor affecting the prognosis of death (OR = 6.68, 95% CI:1.10–40.78, β = 1.90, *P* = 0.040). Receiver operating characteristic (ROC) curve analysis was used to evaluate the predictive value of variables; cut-off values were calculated and the area under the curve was 0.7782 [(95% CI:0.686–0.871), *p <* 0.001] for the prediction of death at 90 days in all patients. The Kaplan-Meier curve used for survival analysis showed that, compared with the normal platelet group, the overall survival rate of patients with low platelet levels was significantly lower, and the difference was statistically significant [HR = 2.11, (95% CI:1.47–3.03), *p* = 0.0001, z = 4.05, X^*2*^ = 14.89]. Cox regression analysis, used to further verify the influence of prognostic risk factors, showed that a concurrent low platelet count was the most important independent risk factor affecting the prognosis of SAP (HR = 2.12 [95% CI:1.12–3.99], X^2^ = 50.95, *p* = 0.021).

**Conclusion:** These findings demonstrate an association between SAP mortality and platelet levels on admission. Thus, platelet level at admission may be used as a readily available marker for assessing the prognosis of patients with SAP.

## 1 Introduction

Pneumonia, which is among the top ten causes of death worldwide, is a commonly occurring infectious disease of the lower respiratory tract. It is categorized into community-acquired pneumonia (CAP) and hospital-acquired pneumonia (HAP), including ventilator-associated pneumonia (VAP; Torres et al., 2021). Epidemiological data show that the incidence of adult community-acquired pneumonia is 5–11·1,000 people^−1^·year^−1^ in European and North American countries (Lim et al., 2009). In the United States, the prevalence of pneumonia is approximately 21% (Eldridge et al., 2022), and a survey from China showed that the overall incidence of CAP was 7.13 [(95% CI:6.11–8.15)] per 1,000 person-years, with more men [7.32 (95% CI:6.28–8.35)] than women [6.93 (95% CI:5.92–7.94)] per 1,000 person-years (Sun et al., 2020). Pneumonia can worsen and develop into a severe form. Research data showed that about one-fifth of hospitalized CAP patients in the United States required intensive care unit (ICU) treatment, with 50% dying within 1 year (Cavallazzi et al., 2020). In China, respiratory diseases rank first in the ICU disease spectrum (21.88%), and pulmonary infections account for the highest proportion of sepsis patients (63.79%; Sun et al., 2022). Therefore, we can infer that severe pneumonia has a huge impact on the social economy and affects the allocation of medical resources. An increasing number of studies show that the occurrence of pneumonia is associated with aspiration. Aspiration pneumonia (AP) refers to pulmonary infectious lesions caused by massive inhalation of oropharyngeal secretions, stomach contents, food, among other objects (Mandell and Niederman 2019). There is no clear definition of AP pathology and no unified diagnostic gold standard (Almirall et al., 2021). Clinically, diagnosis can be made by adding risk factors and/or clinical manifestations of pneumonia in addition to X-ray or chest CT of typical segmental pneumonia-dependent pneumonia (Makhnevich et al., 2019). Our study was based on this as a diagnosis of aspiration pneumonia.

However, as this condition is hidden or silent, it is often ignored by doctors, leading to a missed diagnosis. The common risk factors for aspiration are impaired consciousness, dysphagia, impaired cough reflex, gastroesophageal reflux, or indwelling nasogastric tube/nasointestinal tube. A systematic review published in the New England Journal in 2001 found that aspiration pneumonia accounts for approximately 5%–15% of CAP (Marik 2001), but the proportion of HAP is not clear. According to 2008 survey data (Teramoto et al., 2008) in Japan, AP accounted for 66.8% of hospitalized patients with pneumonia, and the incidence of aspiration in CAP and HAP was 60.1% and 86.7%, respectively. Research data from South Korea showed that the 1-year, 3-year, and 5-year mortality rates of patients with AP were 49.0%, 67.1%, and 76.9%, respectively (Yoon et al., 2019). Compared to non-aspiration pneumonia (non-AP) patients, AP patients seem more serious and have a higher mortality rate (Lindenauer et al., 2018). Several underlying comorbidities increase the level of complexity in ICU patients, such as a long-term bedridden state and endotracheal intubation, all of which are prone to the occurrence of AP. Therefore, it is necessary to focus more on the occurrence of severe aspiration pneumonia (SAP) and its prevention or treatment.

In most cases, pneumonia management requires attention on other aspects, including correct diagnosis of the disease, choice of antibiotics, and treatment sites. The role of markers such as blood parameters or inflammatory indicators has been widely studied in recent years. Traditional bacterial infection biomarkers, such as white blood cells (WBC), erythrocyte sedimentation rate (ESR), neutrophil alkaline phosphatase (NAP) score, C-reactive protein (CRP), and endotoxins, are widely known. Recent researches on the inflammatory biomarker procalcitonin (PCT) and new blood biomarkers, for example, NLR (neutrophilic granulocyte/lymphocyte ratio) and PLR (platelet cell count/lymphocytes) are also being used. A recent multicenter retrospective study in northern Japan found that, compared with non-AP, AP patients have lower BMI (19.4 ± 4.0 vs. 21.4 ± 4.6), C-reactive protein (9.4 ± 8.5 vs. 11.5 ± 9.8 mg/dL), and serum albumin (3.1 ± 0.6 vs. 3.2 ± 0.6) g/dL); higher rates of cerebrovascular disease, dementia and neuromuscular disease complications; and higher mortality rates (Suzuki et al., 2021).

For CAP patients, studies show that NLR levels are elevated and significantly correlated with the Pneumonia Severity Index (PSI) score (Huang et al., 2018). In addition, persistently elevated PCT levels in patients with severe pneumonia are associated with disease progression; therefore, it is a good indicator for evaluating patients’ clinical outcomes (Zheng et al., 2019). Besides blood parameters, age, smoking, environmental leakage, malnutrition, previous pulmonary disease (CAP, chronic obstructive pulmonary disease, bronchial asthma), poor dental health, immunosuppressive drugs, and gastric acid-suppressive drugs are risk factors for pneumonia (Almirall et al., 2017). Fine et al. (Fine et al., 1997) believed that pneumonia patients’ hospitalization rate varied widely, partly due to doctors’ uncertainty associated with assessing the severity of the disease. For this reason, they statistically analyzed the physical examination results, blood parameters, and underlying comorbidities of inpatients and outpatients with pneumonia through a large-scale cohort study. Finally, they obtained a prediction model with a 30-day follow-up time, which could effectively predict the low risk of death and poor prognosis of patients with CAP.

Over the last 2 years, our team members have studied the impact of hematological parameters on the prognosis of ICU patients with AP, but no relevant studies have been reported previously. Therefore, we aim to explore the hematological parameters that can identify the poor prognosis of SAP in ICU patients by analyzing demographic, clinical, radiological, and laboratory data to provide a reference for its treatment plan.

## 2 Materials and methods

### 2.1 Participants

We recruited patients who were admitted to the ICU of Jinshan Hospital, Fudan University, Shanghai, China, for SAP between January 2021 and December 2021. Patients who were 18 years of age or older and met the diagnostic criteria for aspiration pneumonia were included in our study. Exclusions included pneumonia due to a pathogenic bacterial infection combined with other systemic infections and patients with a previous basic history of severe dysfunction of the heart, lung, kidney, and other organs, long-term immunosuppressants or immune dysfunction, and patients who were not suitable for other reasons.

**FIGURE 1:**
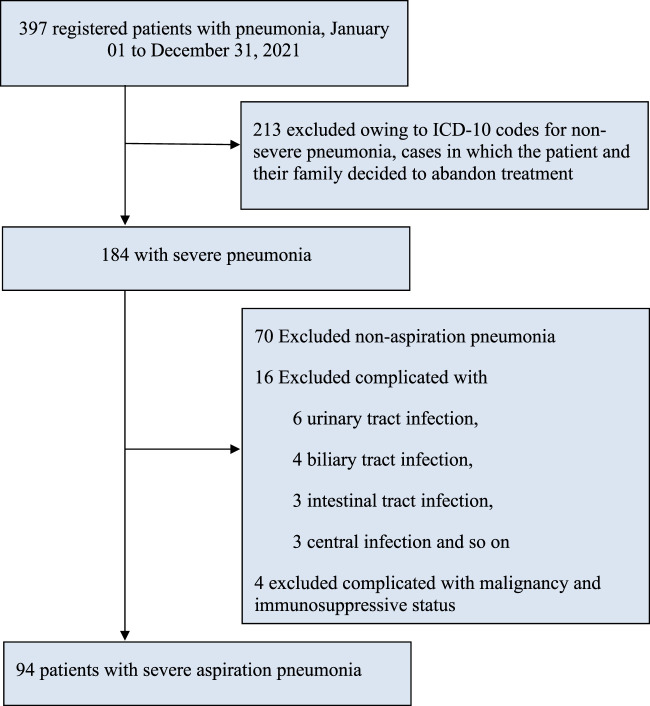
Study flowchart. Note. AP, aspiration pneumonia; ICD-10, International statistical classification of diseases and related health problems, tenth revision; SP, severe pneumonia.

A total of 397 cases were recorded, wherein 213 cases were excluded because of the decision taken by patients and their family members to give up treatment or request for maintenance therapy. In the remaining population (184), we also excluded 70 cases of non-aspiration pneumonia, 6 cases of urinary tract infection, 4 cases of biliary tract infection, 3 cases of intestinal infection, 3 cases of central infection, and 4 cases of malignant tumors and immunosuppressive status, resulting in our study cohort of 94 participants for this analysis (Figure 1).

### 2.2 Study design

This was a retrospective cohort study. Severe pneumonia was diagnosed according to the Guidelines for the Diagnosis and Treatment of Adult Community-Acquired Pneumonia in China (2016 Edition) and the guidelines published in 2007 for CAP by the Infectious Diseases Society of America/American Thoracic Society (Mandell et al., 2007; Qu and Cao, 2016). Direct ICU admission is required for patients with septic shock requiring vasopressors or acute respiratory failure requiring intubation and mechanical ventilation. In addition, we excluded those who were younger than 18 years, critically ill, or died within a short period of time, failed to complete the examination (including lung ultrasound, chest CT scan), suffering from hematological diseases, other malignant tumors, severe immunocompromised disease, severe liver, and kidney disease, and patients with cardiovascular and cerebrovascular diseases.

All the patients were diagnosed based on the presence of aspiration factors, physical examination, and chest imaging. After a 90-day follow up, patients were divided into survival and death groups, consistent with their outcomes.

### 2.3 Data collection

We collected patient information, such as name, sex, age, length of hospital stay, living habits (smoking/drinking), blood routine [WBC, NEUT%, LY%, RBC (red blood cells), HB (hemoglobin), and PLT (platelet)], biochemistry [ALT, TP (total protein), ALB (albumin), GLO (globulin), PA (prealbumin), blood urea nitrogen (BUN), and urea], electrolytes (K+, Na+, Cl−), inflammatory markers (CRP and PCT), vital signs (e.g., temperature, heart rate, respiration, blood pressure), underlying diseases (e.g., cardiovascular and cerebrovascular diseases, diabetes mellitus, liver, and kidney disease), and other data. All laboratory indicators were collected within 24 h after admission, and neutrophil-lymphocyte ratio (NLR), blood cell-to-erythrocyte ratio (WRR), white blood cell count and platelet count ratio (WPR), and platelet-to-white blood cell ratio (PWR) were calculated; meanwhile, we established a database and registered all the data in electronic form.

We collected 6 mL of fasting venous blood from patients in the morning and quickly sent it to the laboratory. The collected blood test items included relevant routine blood indicators, blood biochemistry, C-reactive protein (CRP), and PCT. We used an automatic blood cell analyzer (Micon Sison, Japan) for routine blood tests, Beckman automatic biochemical analyzer AU5800, and a 1,000 biochemical instrument VITROS5600 (Beckman Company, Johnson and Johnson) for blood biochemistry, electrolytes, and blood glucose tests. A CRP Aristo-specific protein analyzer (Shenzhen Guosai Biotechnology Co. LTD., Shenzhen, China) was used to detect CRP concentration. The NLR was calculated based on the absolute neutrophil count divided by the lymphocyte count, WRR was calculated by dividing the white blood cell count by the red blood cell count, WPR was calculated by dividing the white blood cell count by the platelet count, and PWR was calculated by dividing the platelet count by the white blood cell count. Combined with age, gender and weight, CKD-EPI equation was used to calculate glomerular filtration rate (GFR).

### 2.4 Statistical methods

The collected data were analyzed using STATA 15.1 and *p* < 0.05 was considered statistically significant. Normally distributed data are expressed as mean ± standard deviation (x ± s) and Student’s *t*-test was used for making comparisons between the two groups. Data that were not normally distributed were expressed as median (M) and interquartile range (Q1, Q3) and compared using the non-parametric Mann-Whitney *U* test for comparisons between the two groups. The count data were compared using the *χ* ^2^ test. Multiple logistic regression analysis was used to control for confounding factors and evaluate if platelet count could be an independent risk factor for poor prognosis of SAP. To evaluate the sensitivity of platelets in predicting SAP, we performed the receiver operator characteristic (ROC) curve analysis and calculated the area under the curve (AUC) and 95% confidence interval (CI) to compare the prognostic value of different indicators in SAP. The Youden index (Youden index = specificity + sensitivity-1) was used as a reference for clinical classification to determine the optimal cutoff value of platelet indicators for predicting death in patients with SAP. Subsequently, the Kaplan–Meier curves were used to analyze the 90-day survival of patients with different levels of thrombocytopenia. Finally, Cox regression analysis was used to further verify the risk factors affecting the prognosis.

### 2.5 Ethical considerations

This study was reviewed and approved by the Ethics Committee of Jinshan Hospital, Fudan University. Ethical approval number: Jinyi IEC-2020-S25. Chinese Clinical Registration Number: ChiCTR2000035806.

## 3 Results

### 3.1 Baseline characteristics of patients with SAP

Totally, 94 subjects were included in the study cohort, including 66 men (70.21%), and the median (SD) age was 79 years (interquartile range: 69–85 years). According to the clinical outcomes of the 90-day follow-up, patients were divided into a survival group (n = 42) and a death group (n = 52). There were 32 men and 10 women in the survival group, with a median (SD) age of 77.5 years (interquartile range: 67, 82 years), while there were 34 men and 18 women in the death group, with a median age of 81 years (interquartile range: 69.5, 86 years). By comparing information such as gender, age, vital signs, BMI (body mass index), APACH II score (acute physiology score + age points + chronic health points), smoking history, underlying diseases, and laboratory indicators of the two groups, we found that heart rate, suffered coronary heart disease, or liver and kidney diseases, level of white blood cells (WBC), neutrophilic granulocyte percentage (NEUT%), lymphocyte percentage (LY%), neutrophils-to-lymphocytes ratio (NLR), red blood cell (RBC), hemoglobin (HB), white blood cell-to-erythrocyte ratio (WRR), platelets (PLT), white blood cell-to-platelet count ratio (WPR), platelet count-to-white blood cell count ratio (PWR), C-reactive protein (CRP), procalcitonin (PCT), urea, total protein (TP), albumin (ALB), globulin (GLO), prealbumin albumin (PA), brain natriuretic peptide (BNP), BUN, creatinine (Cr), endogenous creatinine clearance rate (Ccr), and serum potassium level (K^+^) were significant differences between the two groups in (*p* < 0.05); however, there were no significant differences in gender, age, body temperature, respiration, blood pressure, BMI, APACH II score, smoking history, combined stroke, cerebral atrophy, hypertension, diabetes mellitus, alanine aminotransferase (ALT), and blood glucose (GLU) between the two groups (*p* > 0.05, Table 1).

**TABLE 1:** Baseline characteristics for patients with SAP.

Characteristics	Patients with severe aspiration pneumonia (*n* = 94)				
	All (*n* = 94)	Survival (*n* = 42)	Died (*n* = 52)	*X* ^*2*^ */t*	*P*
Sex [Male (*n*, %)]	66 (70.21)	32 (76.19)	34 (65.38)	1.23	0.26
	28 (29.79)	10 (23.81)	18 (34.62)		
Age [M(Q1, Q3)] (years)	79 (69, 85)	77.5 (67, 82)	81 (69.5, 86)	2.19	0.14
Vital signs					
Temperature [M(Q1, Q3)] °C	36.8 (36.4, 37.3)	36.7 (36.5, 37)	37 (36.4, 37.5)	1.46	0.22
Heart [M(Q1, Q3)] rate/bpm	99 (88, 113)	91.5 (82, 108)	101 (93.5, 116.5)	5.03	0.025
Respiration [M(Q1, Q3)] rate/bpm	21 (18, 25)	20 (18, 23)	23 (18.5, 28.5)	3.73	0.053
SBP [‾x ± s ] mmHg	129.27 ± 2.67	128.62 ± 4.21	129.79 ± 3.47	−0.21	0.83
DBP [‾x ± s ]mmHg	73.16 ± 1.66	74.55 ± 2.47	72.04 ± 2.25	0.75	0.45
BMI [M(Q1, Q3)] kg/㎡	21.16 (18.03, 24.46)	21.16 (17.72, 24.22)	21.09 (18.21, 25.26)	0.16	0.69
APACH II [M(Q1, Q3)]	22 (19, 25)	22.5 (19, 25)	22 (18.5, 26)	0.095	0.76
Smoking history					
Never smoked [n (%)]	62 (65.96)	29 (69.05)	33 (63.46)	0.59	0.74
Past smoker [n (%)]	19 (20.21)	7 (16.67)	12 (23.08)		
Presently smoking [n (%)]	13 (13.83)	6 (16.67)	7 (13.46)		
Underlying disease					
Stroke [n (%)]	34 (34.04)	13 (30.95)	19 (36.54)	0.32	0.57
Encephalatrophy [n (%)]	24 (25.53)	14 (33.33)	10 (19.23)	2.43	0.12
Hypertension [n (%)]	41 (43.62)	19 (45.24)	22 (42.31)	0.081	0.78
Diabetes [n (%)]	31 (32.98)	16 (38.10)	15 (28.85)	0.90	0.34
Coronary heart disease [n (%)]	52 (55.32)	16 (38.10)	36 (69.23)	9.11	0.003
Lung disease [n (%)]	33 (35.11)	14 (33.33)	19 (36.54)	0.10	0.746
Renal disease [n (%)]	32 (34.04)	9 (21.43)	23 (44.23)	5.38	0.020
Liver disease [n (%)]	21 (22.34)	5 (11.90)	16 (30.77)	4.77	0.029
Laboratory indicators					
WBC [M(Q1, Q3] × 10^9^/L	8.85 (6.5, 13.2)	7.95 (6.00, 9.30)	10.10 (7.65, 17.15)	8.31	0.0039
NEUT% [M(Q1, Q3] %	81.65 (71.6, 91.1)	73.25 (65.4, 82.1)	87.65 (80.85, 94.45)	23.43	0.0001
LY% [M(Q1, Q3] %	10.6 (4.9, 17.4)	16.5 (9.8, 23.6)	7.25 (2.55, 13)	19.93	0.0001
NLR [M(Q1, Q3]	7.66 (4.06, 18.59)	4.56 (2.76, 8.25)	12.11 (6.46, 37.25)	20.55	0.0001
RBC [M(Q1, Q3] × 10^12^/L	3.17 (2.61, 3.69)	3.38 (2.9, 4.01)	3.10 (2.53, 3.38)	8.96	0.0028
HB [M(Q1, Q3] g/L	93 (79, 108)	101.5 (84, 116)	90 (74, 104.5)	5.56	0.0184
WRR [M(Q1, Q3] × 10^−2^	2.73 (1.85, 4.85)	2.12 (1.57, 3.41)	3.83 (2.25, 6.25)	11.71	0.0006
PLT [M(Q1, Q3] × 10^9^/L	164.5 (90, 239)	204 (157, 287)	102.5 (70.5, 180.5)	21.35	0.0001
WPR [M(Q1, Q3] × 10^−2^	6.17 (3.47, 11.06)	3.57 (2.87, 5.43)	10.05 (6.17, 17.88)	32.27	0.0001
PWR [M(Q1, Q3]	16.22 (9.04, 28.80)	28.01 (18.42, 34.89)	9.95 (5.60, 16.22)	32.27	0.0001
CRP [M(Q1, Q3] mg/L	42.49 (11.02, 140.36)	12.60 (6.82, 33.31)	108.43 (42.49, 179.85)	28.18	0.0001
PCT [M(Q1, Q3] ng/mL	0.99 (0.69, 18.92)	0.97 (0.38, 5.14)	5.42 (0.77, 45.52)	6.36	0.0117
ALT [M(Q1, Q3] U/L	37.5 (27, 83)	37.5 (28, 71)	37.5 (23, 107)	0.093	0.7609
TP [‾x ± s ]g/L	54.10 ± 0.96	59.79 ± 1.27	51.12 ± 1.16	5.04	0.000
ALB [‾x ± s] g/L	29.09 ± 0.56	30.90 ± 0.76	27.62 ± 0.75	3.09	0.0026
GLO [‾x ± s] g/L	26.15 ± 0.70	28.88 ± 1.01	23.94 ± 0.87	3.72	0.0004
PA [M(Q1, Q3)] mg/L	105.5 (76, 163)	145.5 (96, 185)	93.5 (67, 133.5)	11.17	00008
BNP [M(Q1, Q3)] pg/mL	126 (38.7, 359.1)	50.7 (18.6, 126)	290.4 (95.9, 510.4)	22.89	0.0001
BUN [M(Q1, Q3)] mmol/L	9.65 (5.7, 17.9)	6.2 (4.5, 9.3)	16.25 (9.5, 24.35)	30.11	0.0001
Cr [M(Q1, Q3)] umol/L	73 (53, 132)	56 (49, 71)	109 (72.5, 253.5)	22.70	0.0001
Uric acid [M(Q1, Q3)] umol/L	264 (199, 335)	253.5 (177, 295)	284.6 (210.5, 511.5)	4.02	0.045
Ccr [M(Q1, Q3)] mL/min	53.1 (30.4, 84.3)	79.9 (52, 93.8)	36.1 (14.2, 63.5)	22.52	0.0001
GLU [M(Q1, Q3)] mmol/L	11.95 (8.07, 15.7)	11.25 (7.84, 15.51)	12.3 (8.49, 16.64)	0.42	0.52
K^+^[M(Q1, Q3)] mmol/L	4.09 (3.73, 4.61)	3.98 (3.73, 4.22)	4.32 (3.77, 4.87)	6.02	0.014
Na^+^[M(Q1, Q3)] mmol/L	141 (136, 145)	140 (136, 142)	142.5 (136, 148)	2.87	0.090
Cl^−^[M(Q1, Q3)] mmol/L	103.5 (99, 108)	102 (100, 107)	105 (99, 108)	0.57	0.45

Note: SBP, systolic blood pressure; DBP, diastolic blood pressure; BMI, body mass index; WBC, white blood cell count; NEUT%, neutrophilic granulocyte percentage; LY%, lymphocyte percentage; NLR, neutrophils to lymphocytes ratio; RBC, red blood cell count; HB, hemoglobin; WRR, white blood cell count to red blood cell count ratio; PLT, platelet count; WPR, white blood cell count to platelet count ratio; PWR, platelet count to white blood cell count ratio; CRP, C-reactive protein; PCT, procalcitonin; ALT, alanine aminotransferase; TP, total protein; ALB, albumin; GLO, globulin; PA, prealbumin; BNP, brain natriuretic peptide; BUN, blood urea nitrogen; Cr, creatinine; Ccr, endogenous creatinine clearance rate; GLU, glucose; K^+^, potassium; Na^+^, Sodium; Cl^−^, Chlorine.

### 3.2 Analysis of risk factors affecting patients’ prognosis

We followed up on all the patients for 90 days, and considered death as the dependent variable, included significant differences in the above-mentioned baseline data and excluded repeated influencing factors. Heart rate, coronary heart disease, liver or kidney disease, WBC, NEUT, HB, PLT, CRP, PCT, BNP, TP, PA, Ccr, and K+ were used as the independent variables to establish a logistic regression model (Supplementary Table S1). We identified coronary heart disease, underlying liver and kidney diseases, WBC, NEUT, PLT, CRP, BNP, TP, PA, and Ccr as the independent variables using univariate logistic analysis. Multivariate logistic regression analysis suggested that PLT (OR = 6.68, 95% CI:1.10–40.78, β = 1.90, *P* = 0.040) was an independent risk factor for SAP (*p* < 0.05) (Table 2).

**TABLE 2:** Results of univariate and multivariate logistic regression analysis.

Varies	Univariate		Multivariate	
	OR (95%)	*p-value*	OR (95%)	*p-value*
HR	0.61 (0.05∼6.97)	0.691	——	——
Coronary heart disease	3.66 (1.55∼8.62)	0.003	3.10 (0.77∼12.41)	0.110
Renal disease	2.91 (1.16∼7.28)	0.023	0.98 (0.21∼4.55)	0.977
Liver disease	3.29 (1.09∼9.92)	0.035	1.08 (0.17∼6.87)	0.935
WBC	4.36 (1.78∼10.71)	0.001	0.88 (0.14∼5.34)	0.886
NEUT%	10.50 (4.02∼27.45)	0.000	5.71 (0.93∼35.17)	0.060
HB	1.62 (0.94∼2.77)	0.081	——	——
PLT	13.89 (3.13∼61.73)	0.001	6.68 (1.10∼40.78)	0.040
CRP	1.86 (1.46∼2.37)	0.000	1.21 (0.81∼1.82)	0.353
PCT	1.65 (0.98∼2.81)	0.062	——	——
BNP	3.94 (2.00∼7.76)	0.000	1.95 (0.60∼12.53)	0.244
TP	7.07 (2.60∼19.23)	0.000	2.75 (0.60∼12.53)	0.192
PA	1.98 (1.30∼2.99)	0.001	0.73 (0.33∼1.63)	0.444
Ccr	2.39 (1.58∼3.60)	0.000	1.37 (0.78∼2.44)	0.270
K+	1.01 (0.25∼4.03)	0.988	——	——

Note: HR, heart rate; WBC, white blood cell count; NEUT%, neutrophilic granulocyte percentage; HB, hemoglobin; PLT, platelet count; CRP, C-reactive protein; PCT, procalcitonin; BNP, brain natriuretic peptide; TP, total protein; PA, prealbumin; Ccr, endogenous creatinine clearance rate; K^+^, potassium.

### 3.3 Analysis of the predictive value of blood routine-related indicators, CRP, and PCT on the risk of death in SAP

**FIGURE 2:**
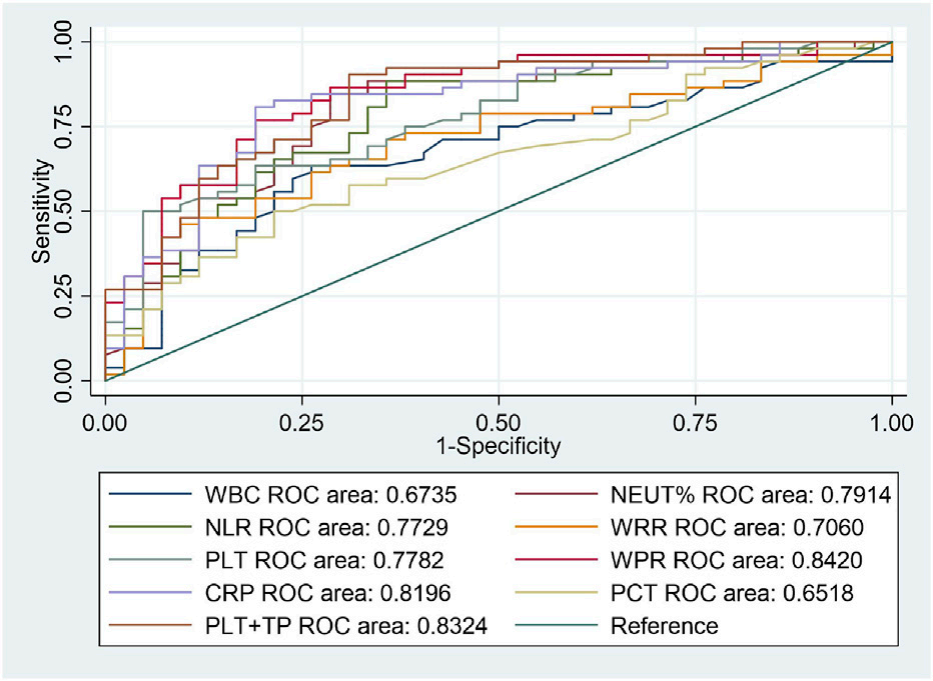
ROC curves of blood routine indexes, CRP, and PCT predicting the 90-day death prognosis of patients with SAP.

From the above results, we can conclude that PLT is an independent risk factor for SAP (*p* < 0.05). However, the relationship between other routine blood indicators and platelets remains unclear. We further investigated the predictive value of WBC count, NEUT%, NLR, WPR, PLT, WRR, CRP, and PCT for assessing the risk of death due to SAP. We performed ROC curve analysis on the blood routine-related indicators CRP and PCT for patients with SAP. The analysis results showed that the AUCs of WBC, NEUT%, NLR, WPR, PLT, WRR, CRP, and PCT were 0.6735, 0.7914, 0.7729, 0.842, 0.7782, 0.706, 0.8196, and 0.6518, respectively, which could effectively predict the occurrence of death (*p* < 0.05) (Figure 2). After sorting, we found that the order from highest to lowest was WPR  >  PLT + TP  >  CRP > NEUT% >  PLT > NLR > WRR > WBC > PCT. Moreover, we further analyzed the data statistically, and the results showed that the best cutoff value of PLT was 127 × 10^9^/L, with a sensitivity of 63.46% and a specificity of 80.95%. When it combined platelet to calculate the AUC, we found out that the AUC of the combined index was 0.8324, with the sensitivity and specificity were 90.38% and 69.05% respectively (Table 3). The sensitivity, specificity, and optimal cutoff values of other indicators are listed in Table 3.

**TABLE 3:** Detailed report of cutpoint, sensitivity, and specificity.

Varies	Cutpoint	Sensitivity (%)	Specificity (%)	CorrectlyClassified (%)	LR+	LR-
WPR	4.36	86.54	71.43	79.79	3.0288	0.1885
PLT + TP	0.41	90.38	69.05	80.85	2.9201	0.1393
CRP	40.47	80.77	80.95	80.85	4.2404	0.2376
NEUT%	78.10	84.62	71.43	78.72	2.9615	0.2154
PLT	127	63.46	80.95	71.28	3.3317	0.4514
NLR	5.05	88.46	64.29	77.66	2.4769	0.1795
WRR	2.49	73.08	61.90	68.09	1.9183	0.4349
WBC	9.2	63.46	73.81	68.09	2.4231	0.4950
PCT	5.69	50.00	78.57	62.77	2.3333	0.6364

Note: +LR, positive likelihood ratio; –LR, negative likelihood ratio; WPR, white blood cell count to platelet count ratio; PLT, platelet count; TP, total protein; CRP, C-reactive protein; NEUT%, neutrophilic granulocyte percentage; NLR, neutrophils to lymphocytes ratio; WRR, white blood cell count to red blood cell count ratio; WBC, white blood cell count; PCT, procalcitonin.

### 3.4 Analysis of short-term survival among hospitalized patients with SAP and with or without low level platelets

The survival curves were compared using the Kaplan-Meier method to evaluate the short-term survival of SAP, and Cox regression was used for survival analysis. The hazard ratio (HR) does not change with time; that is, it meets the proportional hazards assumption (PH assumption). The observed curve is the Kaplan-Meier curve obtained from actual data, and the predicted curve is the curve fitted by the Cox model, assuming that PH is established. The curves almost coincide in the groups with different platelet levels. Therefore, the PH hypothesis is valid.

**FIGURE 3:**
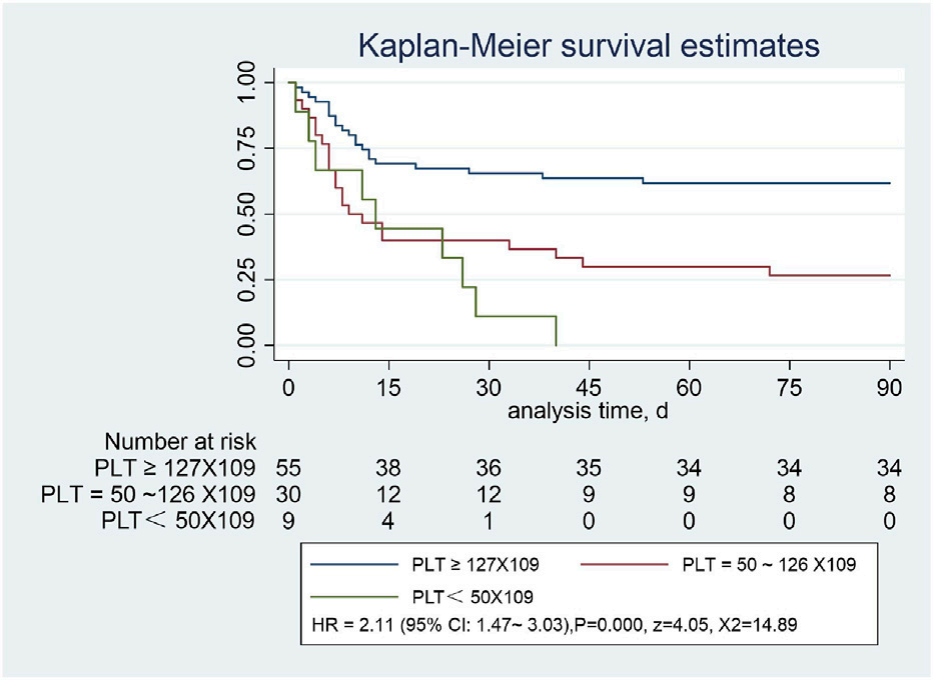
Severe pneumonia with or without low platelet count, assessed using the Kaplan-Meier estimator.

We plotted the Kaplan-Meier curves and performed the log-rank test. In addition, HR was analyzed using Cox regression analysis approach. In this study, we followed all the patients for 90 days. Patients with SAP were divided into the normal platelet group (≥127 × 10^9^/L), lower platelet level group (50-126*10^9^/L), and lowest platelet level group (<50 × 10^9^/L). The log-rank test showed a statistically significant difference between the three groups (X^*2*^ = 18.22, *p* = 0.0001). The overall survival rates of the three groups were completely different. In contrast, the overall survival rate of the low platelet level group was significantly lower than that of the normal platelet group. Statistically significant differences were observed in the 90-day survival curves among the three groups [HR = 2.11, 95% CI:1.47–3.03), *p = 0.0001,* z = 4.05, X^*2*^ = 14.89] (Figure 3).

### 3.5 Cox multivariate regression analysis on the prognosis of SAP

We analyzed the all-cause mortality risk using the Cox proportional method. Entering each variable into the Cox model, the multivariate analysis of the affecting SAP suggested that thrombocytopenia was the highly important independent risk factor affecting the prognosis of SAP [HR = 2.12 (95% CI:1.12–3.99), X^*2*^ = 50.95, *p* = 0.021]; otherwise, we found that the reduction in total protein levels is also a major risk factor affecting SAP prognosis (Table 4).

**TABLE 4:** Cox proportional analysis of all-cause mortality risk regression, showing the results of the adjusted analysis.

Varies	Multivariate	
	HR (95%)	*p-value*
Coronary heart disease	1.67 (0.88∼3.17)	0.114
Renal disease	1.22 (0.60∼2.49)	0.583
Liver disease	0.85 (0.39∼1.85)	0.681
WBC	0.88 (0.46∼1.70)	0.704
NEUT%	2.40 (0.98∼5.86)	0.055
PLT	2.12 (1.12∼3.99)	0.021
CRP	1.11 (0.87∼1.42)	0.410
BNP	1.07 (0.66∼1.73)	0.790
TP	2.67 (1.07∼6.67)	0.035
PA	0.90 (0.61∼1.33)	0.599
Ccr	1.13 (0.87∼1.50)	0.352

Note: WBC, white blood cell count; NEUT%, neutrophilic granulocyte percentage; PLT, platelet count; CRP, C-reactive protein; BNP, brain natriuretic peptide; TP, total protein; PA, prealbumin; Ccr, endogenous creatinine clearance rate.

## 4 Discussion and conclusion

Pneumonia is an age-related disease and a common cause of hospitalization in the elderly population. With the acceleration of the global aging process, the elderly population is gradually increasing, and attention should be paid to their health problems (Cillóniz et al., 2018). In Japan, the incidence of CAP among patients aged 65–74 years and those aged 75 years or older is 10.7·1,000-1·year-1 and 42.9·1,000-1·year-1, respectively (Takaki et al., 2014), while Chinese research data showed that CAP patients aged over 65 years accounted for as much as 28.7% of the population (Liu et al., 2013). The occurrence of aspiration pneumonia in the elderly is related to smoking, underlying diseases, and medication history; therefore, attention should be paid to assessing the risk factors for its occurrence and formulating corresponding preventive measures. Aspiration pneumonia was considered a subclass of CAP (Rodriguez and Restrepo 2019), with a high severity of disease and a higher risk of poor prognosis of death than non-AP. The common pathogen spectrum of severe pneumonia includes bacteria, fungi, viruses, and parasites, which often aggravate infection. Fleur P team member's research showed that the risk of severe pneumonia was 4 times higher in patients with *S. aureus* colonized at ICU admission than in those without (Paling et al., 2020); therefore, microbial suction and engraftment are the cause of pneumonia illness weight, and this may be related to intensive care unit patients with complex flora, illness weight, length of hospital stay (days), and airway opening. Therefore, timely identification of the severity and grade of pneumonia, early intervention, pneumonia treatment site, and choice of antibiotics have important guiding significance.

Over the past few decades, an increasing number of studies have been conducted on blood parameters that are crucial in the role of inflammatory responses. As an important blood component of the human body, platelets are small pieces of cytoplasm that are lysed from the cytoplasm of mature megakaryocytes in the bone marrow. They play an active role in a variety of physiological reactions and in maintaining blood coagulation, and participate in the regulation of tumor growth, metastasis, inflammation, infection, and immune response. Many biological functions and mechanisms of platelets have not yet been studied or understood, which may guide future research and have certain application prospects (Van der Meijden and Heemskerk 2019). The role of platelets has been widely studied in conditions other than hematological diseases, including sepsis (Vardon Bounes et al., 2018), chronic obstructive pulmonary disease (Skoczyński et al., 2019), cardiovascular disease (Feldman and Anderson 2020), cancer (Schlesinger 2018; Plantureux et al., 2020), neurodegenerative diseases (Ferrer-Raventós and Beyer 2021), and tuberculosis (Kirwan et al., 2021). The etiology and pathogenesis of thrombocytopenia are complex and include decreasing platelet production, increasing consumption and destruction, abnormal distribution, and hemodilution. Primary thrombocytopenia is usually found in chronic diseases of the blood system, while acquired thrombocytopenia is found in infections, trauma, surgery, drugs, radiotherapy, immune dysfunction, and nutritional disorders. A study of emergency-related thrombocytopenia showed that its main causes included liver cirrhosis, hematological tumors, chemotherapy-induced thrombocytopenia (CIT), disseminated intravascular coagulation (DIC), infection, and drug-mediated thrombocytopenia (Turvani et al., 2014). Infection is one of the causes of thrombocytopenia, in which platelets are mobilized to the blood vessel wall in an inflammatory response, interacting with white blood cells, allowing leukemia to aggregate to the site of inflammation, appearing to plug fissures in the blood vessel wall, and helping to maintain the integrity of blood vessels (Ho-Tin-Noé et al., 2018).

Although we currently lack direct evidence, it has been suggested that the lungs are an important site for platelet production, which is regulated by inflammation, infection, and lung pathology (Lefrançais and Looney 2019). The results of our multivariate logistic regression analysis showed that only thrombocytopenia was an independent risk factor for the prognosis of patients with SAP (OR = 6.68, 95%CI:1.10–40.78, *β* = 1.90, *p* = 0.040). The area under the ROC curve was 0.7782, with a sensitivity of 63.46% and a specificity of 80.95%, and the best cut-off value was 127 × 10^9^/L, suggesting that we should be alert to the risk of death and other poor prognoses in patients with SAP when platelets are lower than 127 × 10^9^/L. Considering that platelets were obtained through routine extraction from blood, we further compared the differences of some blood routine indexes and inflammatory indexes between the survival group and the death group, and found that there were statistically significant differences in WBC, NEUT%, NLR, WPR, PLT, WRR, CRP, and PCT between the two groups (*p* < 0.05); moreover, the ROC curve analysis showed that the AUC area of WPR was the largest. Leukocytes and platelets are also involved in inflammation. Leukocyte and platelet functions are similar when considering growth factor releasing (Puidokas et al., 2019); therefore, WPR is also a good indicator of the degree of inflammation.

In this study, Kaplan-Meier curves showed a significant reduction in overall survival in patients with significantly reduced platelet counts compared with those with normal platelets (*p* < 0.001). When PLT was <50 × 10^9^/L, all the enrolled patients died within 45 days, and the COX multivariate regression analysis showed that low platelet levels were the most important independent risk factor affecting the prognosis of SAP (HR = 2.12 [95%CI:1.12–3.99*,* X2 = 50.95, *p =* 0.000). We speculate that this may be related to the massive consumption of PLT in the inflammatory response, and its predictive value should not be ignored. Cho et al. (2020) found that the PLT level in the death group was significantly lower than that in the survival group (*p* < 0.001), and the mean platelet volume to platelet count ratio (MPR) was positively correlated with the 60-day patient mortality; that is, higher MPR values were associated with higher mortality risk, which was consistent with our study. Platelets are widely involved in inflammatory responses in addition to their roles in hemostasis and maintenance of vessel wall integrity; the mechanisms of interaction between platelets and bacteria are of increasing interest, and platelet receptors and other host molecules can be used to develop new therapeutic strategies (Yadav et al., 2019). A systematic review (Yeung et al., 2018) showed that in addition to traditional therapeutic targets that inhibit platelet activity, such as cyclooxygenase-1, integrin αIIb β3, and the P2Y 12 receptor, signaling pathways that regulate platelet function (e.g., G protein–coupled receptors, integrin receptors αIIb β3, α2β1, immunoreceptor tyrosine-based activation motif receptors, and enzymes targeted for the regulation of platelet function) dealed with platelet-related pathologies by inhibiting platelet reactivity. Our study suggests that the value of thrombocytopenia in predicting 90-day mortality in SAP warrants further study. Since platelets are an easy-to-obtain and monitor indicator in routine blood, future research can target platelet levels to provide new targets for the prevention and treatment of SAP. Moreover, we found that a decrease in total protein level is also a risk factor affecting the prognosis of SAP. It is speculated that this may be related to the weakening of the body caused by nutritional consumption, which further aggravates poor prognosis; however, further experimental research is needed to verify this.

In conclusion, our findings suggest that there is a correlation between SAP mortality and platelet levels on admission, and the lower the platelet level on admission is, the higher the risk of death is.

## 5 Limitations and innovations

Our study has some limitations. This was a retrospective, single-center observational study with a sample size of only 94 subjects. Therefore, a large-sample, multicenter, randomized controlled trial is required for further verification and generalizability.

This study's innovation exists in identification of the prognostic value of platelets for SAP. Besides, this indicator can be easily obtained, for nearly every medical institution can collect platelets. Therefore, the value of platelet in SAP should not be ignored.

## Data Availability

The original contributions presented in the study are included in the article/Supplementary Material, further inquiries can be directed to the corresponding author.

## References

[B1] Almirall J. Boixeda R. de la Torre M. C. Torres A. (2021). Aspiration pneumonia: A renewed perspective and practical approach. Respir. Med. 185, 106485. 10.1016/j.rmed.2021.106485 34087609

[B2] Almirall J. Serra-Prat M. Bolíbar I. Balasso V. (2017). Risk factors for community-acquired pneumonia in adults: A systematic review of observational studies. Respiration 94, 299–311. 10.1159/000479089 28738364

[B3] Cavallazzi R. Furmanek S. Arnold F. W. Beavin L. A. Wunderink R. G. Niederman M. S. (2020). The burden of community-acquired pneumonia requiring admission to ICU in the United States. Chest 158, 1008–1016. 10.1016/j.chest.2020.03.051 32298730 PMC9458541

[B4] Cho J. Lee S. Uh Y. Lee J. H. (2020). Usefulness of mean platelet volume to platelet count ratio for predicting the risk of mortality in community-acquired pneumonia. Arch. Med. Sci. 16, 1327–1335. 10.5114/aoms.2020.92404 33224331 PMC7667432

[B5] Cillóniz C. Rodríguez-Hurtado D. Torres A. (2018). Characteristics and management of community-acquired pneumonia in the era of global aging. Med. Sci. (Basel). 6, 35. 10.3390/medsci6020035 29710871 PMC6024853

[B6] Eldridge N. Wang Y. Metersky M. Eckenrode S. Mathew J. Sonnenfeld N. (2022). Trends in adverse event rates in hospitalized patients, 2010–2019. JAMA 328, 173–183. 10.1001/jama.2022.9600 35819424 PMC9277501

[B7] Feldman C. Anderson R. (2020). Platelets and their role in the pathogenesis of cardiovascular events in patients with community-acquired pneumonia. Front. Immunol. 11, 577303. 10.3389/fimmu.2020.577303 33042161 PMC7527494

[B8] Ferrer-Raventós P. Beyer K. (2021). Alternative platelet activation pathways and their role in neurodegenerative diseases. Neurobiol. Dis. 159, 105512. 10.1016/j.nbd.2021.105512 34537329

[B9] Fine M. J. Auble T. E. Yealy D. M. Hanusa B. H. Weissfeld L. A. Singer D. E. (1997). A prediction rule to identify low-risk patients with community-acquired pneumonia. N. Engl. J. Med. 336, 243–250. 10.1056/NEJM199701233360402 8995086

[B10] Ho-Tin-Noé B. Boulaftali Y. Camerer E. (2018). Platelets and vascular integrity: How platelets prevent bleeding in inflammation. Blood 131, 277–288. 10.1182/blood-2017-06-742676 29191915

[B11] Huang Y. Liu A. Liang L. Jiang J. Luo H. Deng W. (2018). Diagnostic value of blood parameters for community-acquired pneumonia. Int. Immunopharmacol. 64, 10–15. 10.1016/j.intimp.2018.08.022 30144639

[B12] Kirwan D. E. Chong D. L. W. Friedland J. S. (2021). Platelet activation and the immune response to tuberculosis. Front. Immunol. 12, 631696. 10.3389/fimmu.2021.631696 34093524 PMC8170316

[B13] Lefrançais E. Looney M. R. (2019). Platelet biogenesis in the lung circulation. Physiol. (Bethesda) 34, 392–401. 10.1152/physiol.00017.2019 PMC6957358 31577166

[B14] Lim W. S. Baudouin S. V. George R. C. Hill A. T. Jamieson C. Le Jeune I. (2009). BTS guidelines for the management of community acquired pneumonia in adults: Update 2009. Thorax 64 (3), iii1–ii55. 10.1136/thx.2009.121434 19783532

[B15] Lindenauer P. K. Strait K. M. Grady J. N. Ngo C. K. Parisi M. L. Metersky M. (2018). Variation in the diagnosis of aspiration pneumonia and association with hospital pneumonia outcomes. Ann. Am. Thorac. Soc. 15, 562–569. 10.1513/AnnalsATS.201709-728OC 29298090

[B16] Liu H. Xiao X. C. Lu J. Y. Chen Z. Q. Luo L. Yang Z. C. (2013). Study on epidemic characteristics and etiology of community acquired pneumonia in Guangzhou from 2009 to 2012. Chin. J. Prev. Med. 47, 1089–1094. 10.3760/cma.j.issn.0253-9624.2013.12.005 24529265

[B17] Makhnevich A. Feldhamer K. H. Kast C. L. Sinvani L. (2019). Aspiration pneumonia in older adults. J. Hosp. Med. 14 (7), 429–435. 10.12788/jhm.3154 30794136

[B18] Mandell L. A. Niederman M. S. (2019). Aspiration pneumonia. N. Engl. J. Med. 380, 651–663. 10.1056/NEJMra1714562 30763196

[B19] Mandell L. A. Wunderink R. G. Anzueto A. Bartlett J. G. Campbell G. D. Dean N. C. (2007). Infectious Diseases Society of America/American Thoracic Society consensus guidelines on the management of community-acquired pneumonia in adults. Clin. Infect. Dis. 44 (2), S27–S72. 10.1086/511159 17278083 PMC7107997

[B20] Marik P. E. (2001). Aspiration pneumonitis and aspiration pneumonia. N. Engl. J. Med. 344, 665–671. 10.1056/NEJM200103013440908 11228282

[B21] Paling F. P. Hazard D. Bonten M. J. M. Goossens H. Jafri H. S. Malhotra-Kumar S. (2020). Association of *Staphylococcus aureus* colonization and pneumonia in the intensive care unit. JAMA Netw. Open. 3, e2012741. 10.1001/jamanetworkopen.2020.12741 32997125 PMC7527877

[B22] Plantureux L. Mège D. Crescence L. Carminita E. Robert S. Cointe S. (2020). The interaction of platelets with colorectal cancer cells inhibits tumor growth but promotes metastasis. Cancer Res. 80, 291–303. 10.1158/0008-5472.CAN-19-1181 31727628

[B23] Puidokas T. Kubilius M. Stumbras A. Juodzbalys G. (2019). Effect of leukocytes included in platelet concentrates on cell behaviour. Platelets 30, 937–945. 10.1080/09537104.2019.1646900 31340699

[B24] Qu J. M. Cao B. (2016). [Guidelines for the diagnosis and treatment of adult community acquired pneumonia in China (2016 Edition)]. Chin. J. Tuberc. Respir. Dis. 39, 241–242. 10.3760/cma.j.issn.1001-0939.2016.04.001 27117069

[B25] Rodriguez A. E. Restrepo M. I. (2019). New perspectives in aspiration community acquired Pneumonia. Expert Rev. Clin. Pharmacol. 12, 991–1002. 10.1080/17512433.2019.1663730 31516051

[B26] Schlesinger M. (2018). Role of platelets and platelet receptors in cancer metastasis. J. Hematol. Oncol. 11, 125. 10.1186/s13045-018-0669-2 30305116 PMC6180572

[B27] Skoczyński S. Krzyżak D. Studnicka A. Ogonowski M. Tobiczyk E. Brożek G. (2019). Chronic obstructive pulmonary disease and platelet count. Adv. Exp. Med. Biol. 1160, 19–23. 10.1007/5584_2019_379 31049844

[B28] Sun M. Wu T. Tian H. (2022). [Analysis of disease composition and outcome of patients in intensive care department of 3A hospitals: Analysis of 3 249 cases in the department of liaocheng people’s hospital from 2019 to 2021]. Zhonghua Wei Zhong Bing Ji Jiu Yi Xue 34, 183–187. 10.3760/cma.j.cn121430-20220113-00057 35387726

[B29] Sun Y. Li H. Pei Z. Wang S. Feng J. Xu L. (2020). Incidence of community-acquired pneumonia in urban China: A national population-based study. Vaccine 38, 8362–8370. 10.1016/j.vaccine.2020.11.004 33199077

[B30] Suzuki J. Ikeda R. Kato K. Kakuta R. Kobayashi Y. Ohkoshi A. (2021). Characteristics of aspiration pneumonia patients in acute care hospitals: A multicenter, retrospective survey in northern Japan. PLoS One 16 (7), e0254261. 10.1371/journal.pone.0254261 34329339 PMC8323917

[B31] Takaki M. Nakama T. Ishida M. Morimoto H. Nagasaki Y. Shiramizu R. (2014). High incidence of community-acquired pneumonia among rapidly aging population in Japan: A prospective hospital-based surveillance. Jpn. J. Infect. Dis. 67, 269–275. 10.7883/yoken.67.269 25056072

[B32] Teramoto S. Fukuchi Y. Sasaki H. Sato K. Sekizawa K. Matsuse T. (2008). High incidence of aspiration pneumonia in community- and hospital-acquired pneumonia in hospitalized patients: A multicenter, prospective study in Japan. J. Am. Geriatr. Soc. 56, 577–579. 10.1111/j.1532-5415.2008.01597.x 18315680

[B33] Torres A. Cilloniz C. Niederman M. S. Menéndez R. Chalmers J. D. Wunderink R. G. (2021). Pneumonia. Nat. Rev. Dis. Prim. 7, 25. 10.1038/s41572-021-00259-0 33833230

[B34] Turvani F. Pigozzi L. Barutta L. Pivetta E. Pizzolato E. Morello F. (2014). Bleeding prevalence and transfusion requirement in patients with thrombocytopenia in the emergency department. Clin. Chem. Lab. Med. 52, 1485–1488. 10.1515/cclm-2014-0224 24815053

[B35] Van der Meijden P. E. J. Heemskerk J. W. M. (2019). Platelet biology and functions: New concepts and clinical perspectives. Nat. Rev. Cardiol. 16, 166–179. 10.1038/s41569-018-0110-0 30429532

[B36] Vardon Bounes F. Mujalli A. Cenac C. Severin S. Le Faouder P. Chicanne G. (2018). The importance of blood platelet lipid signaling in thrombosis and in sepsis. Adv. Biol. Regul. 67, 66–73. 10.1016/j.jbior.2017.09.011 28993230

[B37] Yadav V. K. Singh P. K. Agarwal V. Singh S. K. (2019). Crosstalk between platelet and bacteria: A therapeutic prospect. Curr. Pharm. Des. 25, 4041–4052. 10.2174/1381612825666190925163347 31553286

[B38] Yeung J. Li W. Holinstat M. (2018). Platelet signaling and disease: Targeted therapy for thrombosis and other related diseases. Pharmacol. Rev. 70, 526–548. 10.1124/pr.117.014530 29925522 PMC6013590

[B39] Yoon H. Y. Shim S. S. Kim S. J. Lee J. H. Chang J. H. Lee S. H. (2019). Long-term mortality and prognostic factors in aspiration pneumonia. J. Am. Med. Dir. Assoc. 20, 1098–1104. 10.1016/j.jamda.2019.03.029 31080159

[B40] Zheng G. Zhang J. Yuan Y. Xu D. Dong S. Wang H. (2019). Application value of procalcitonin clearance rate on clinical outcome in patients with severe pneumonia. Zhonghua Wei Zhong Bing Ji Jiu Yi Xue 31, 566–570. 10.3760/cma.j.issn.2095-4352.2019.05.009 31198141

