# Supplementary material for: Early platelet level reduction as a prognostic factor in intensive care unit patients with severe aspiration pneumonia
Source: Front Physiol. 2023 Mar 7;14:1064699. doi: 10.3389/fphys.2023.1064699 (PMC10029141; doi:10.3389/fphys.2023.1064699)
Supplement: Supplementary file 1 [file Table1.DOCX]

**Suppl. Table 2**. Description of Variable Assignment.

| **Variables** | **Factors** | **Assignment Description** |
| --- | --- | --- |
| ***X1*** | HR times/min | <100=0; ≥100=1 |
| ***X2*** | Coronary heart disease | No=0; Yes=1 |
| ***X3*** | Renal disease | No=0; Yes=1 |
| ***X4*** | Liver disease | No=0; Yes=1 |
| ***X5*** | WBC (×10^9^/L ) | 4~10=0; <4 or >10=1 |
| ***X6*** | NEUT%(%) | <80=0, ≥80=1 |
| ***X7*** | HB(g/L) | ≥120=0; 119~90=1; 89~60=2; <60=3 |
| ***X8*** | PLT (×10^9^/L ) | ≥100×10^9^/L =0; 99~50=1; 49~20=2; <20=3 |
| ***X9*** | CRP(mg/L) | <10=0; 0~19=1;20~29=2;30~39=3;40~49=4;≥50=5 |
| ***X10*** | PCT(ng/L) | <0.5=0; 0.5~1.9=1;≥2=2 |
| ***X11*** | BNP(pg/ml) | <100=0；100~399=1; ≥400=2 |
| ***X12*** | TP(g/L) | ≥60=0; <60=1 |
| ***X13*** | PA(g/L) | ≥200=0; 199~150=1; 149~100=2; <100=3 |
| ***X14*** | Ccr(ml/min) | ≥80=0; 79~50=1; 49~30=2; 29~20=3;<20=4 |
| ***X15*** | K^+^(mmol/L) | 3.5~5.0=1; <3.5 or >5.0=1 |
| ***Y*** | Mortality | NO=0; Yes=1 |

*Note.* HR=heart rate; WBC=white blood cell count; NEUT%=[neutrophilic granulocyte](http://dict.youdao.com/w/eng/neutrophilic_granulocyte/#keyfrom=dict.basic.syno) percentage; RBC=red blood cell count; HB=hemoglobin; PLT=platelet count; CRP=C-reactive protein; PCT=procalcitonin; BNP=brain natriuretic peptide; TP=total protein; PA=prealbumin; Ccr=endogenous creatinine clearance rate; K^+^=Potassium.
